# O anus, where art thou? An investigation of ctenostome bryozoans

**DOI:** 10.1002/jmor.21146

**Published:** 2020-06-16

**Authors:** Thomas Schwaha

**Affiliations:** ^1^ Department of Evolutionary Biology University of Vienna Vienna Austria

**Keywords:** colonial integration, defecation, soft body morphology, vestibular anus

## Abstract

Ctenostome bryozoans are a small group of approximately 350 currently described species that remain inadequately investigated anatomically. Recently, the importance of soft body morphology of zooids including the digestive tract has become more evident for addressing various biological aspects such as systematic, functional, or phylogenetic analyses. Particularly, the position of the anus shows considerable variation in ctenostomes and in its extreme form can either be at the lophophoral base or at the vestibular wall. However, it has never been analysed in a broader systematic, phylogenetic, or functional context. Hence, the purpose of this study is to assess the distribution of anus position among ctenostomes, analyse whether zooidal or colonial morphology affects anus position, and draw first conclusions on its functional effects. The survey shows that a vestibular anus is ubiquitously present in alcyonidioideans and several, probably closely related, walkerioideans. In other groups such as boring forms, it appears more patchily distributed, or in some currently unassignable genera, such as *Monobryozoon*, supports a closer relationship to alcyonidioideans. Other zooidal or colonial characters such as tentacle number or zooidal density in the colony do not show a distinct correlation to the position of the anus. It appears that the shift of the anus into a vestibular area occurred once or twice among ctenostomes; the reasons and functional effects remain unknown. Future important aspects of defecation research in bryozoans are discussed.

## Graphical abstract

Extended and retracted bryozoan zooids with lophophoral and vestibular anus.

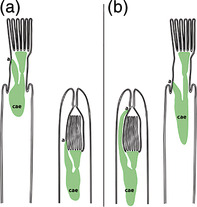

## 1 INTRODUCTION

Coloniality is a key character of the phylum Bryozoa. Colonies are composed of iterated modules called zooids that, in their original form, are represented entirely by autozooids which can feed on their own (Ryland, 1970; Schack, Gordon, & Ryan, 2019). Zooids are traditionally divided into the cystid, which is the protective body wall, and the polypide, which comprises major organ systems such as the tentacle crown (lophophore) used for creating ciliary feeding currents, the U‐shaped digestive tract and associated muscular and neural tissue (Mukai, Terakado, & Reed, 1997; Schwaha, Ostrovsky, & Wanninger, 2020).

**FIGURE 1:**
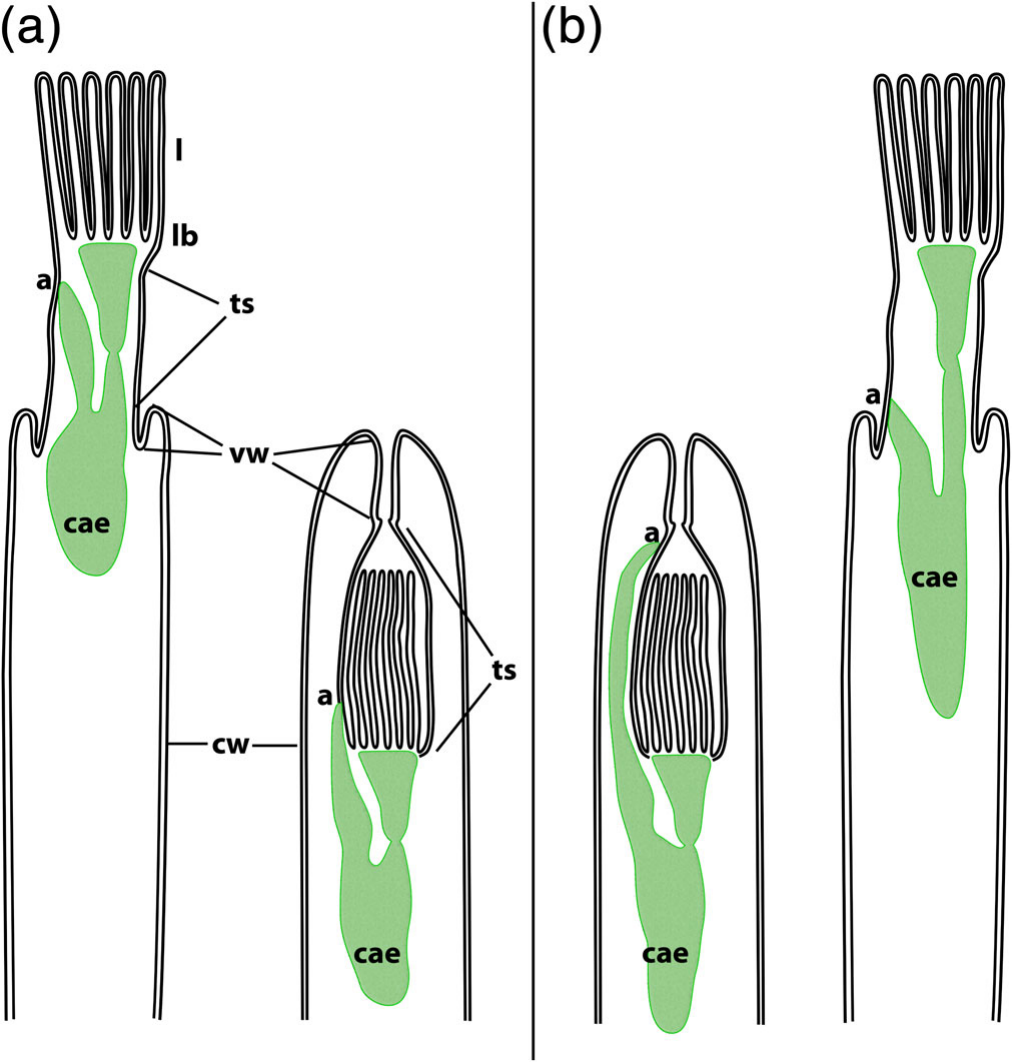
Schematic drawing of the lophophoral (a) and vestibular anus (b) in protruded and contracted condition. The digestive tract is generalised and does not reflect true conditions of the proportions of most bryozoans (mostly due to the lack of comparative data on gut anatomy). Abbreviations: a, anus; cae, caecum; cw, cystid wall; l, lophophore; lb, lophophoral base; ts, tentacle sheath; vw, vestibular wall

A distinct feature, a defensive mechanism, present in all bryozoans is the retractability of the polypide into the cystid. This is achieved by prominent retractor muscles that pull the soft tissues into the protective body wall, versus the protrusion mechanism that involves body‐wall musculature (or its derivatives) to increase hydrostatic pressure within the zooid to squeeze out the polypide enabling it to filter‐feed again (Taylor, 1981). The retraction process causes the introversion of the tentacle sheath, which is a thin body wall connecting the lophophoral base with the cystid wall (Figure 1). The tentacle sheath thus wraps around the lophophore when zooids are retracted. In addition, the vestibular wall, which connects the tentacle sheath to the remaining body wall, can also be highly introvertable (Schwaha, 2019a).

The digestive tract of bryozoans is U‐shaped and divided into three distinct areas: foregut, midgut, and hindgut. The latter terminates via the anus in the tentacle sheath (Silén, 1944, Schwaha et al., 2020, see also Figure 1).

Two large clades of bryozoans can be distinguished—Phylactolaemata and Myolaemata: the latter comprising the Stenolaemata and Gymnolaemata (Schwaha et al., 2020). Phylactolaemates are a small group of freshwater bryozoans. Stenolaemates are an evolutionarily old taxon with only the Cyclostomata being present in recent times and gymnolaemates are the largest with over 5.000 described species (Taylor & Waeschenbach, 2015). This clade can be divided into the paraphyletic ctenostomes and the monophyletic Cheilostomata, which are calcified and the largest taxon of bryozoans (Taylor & Waeschenbach, 2015; Todd, 2000).

**FIGURE 2:**
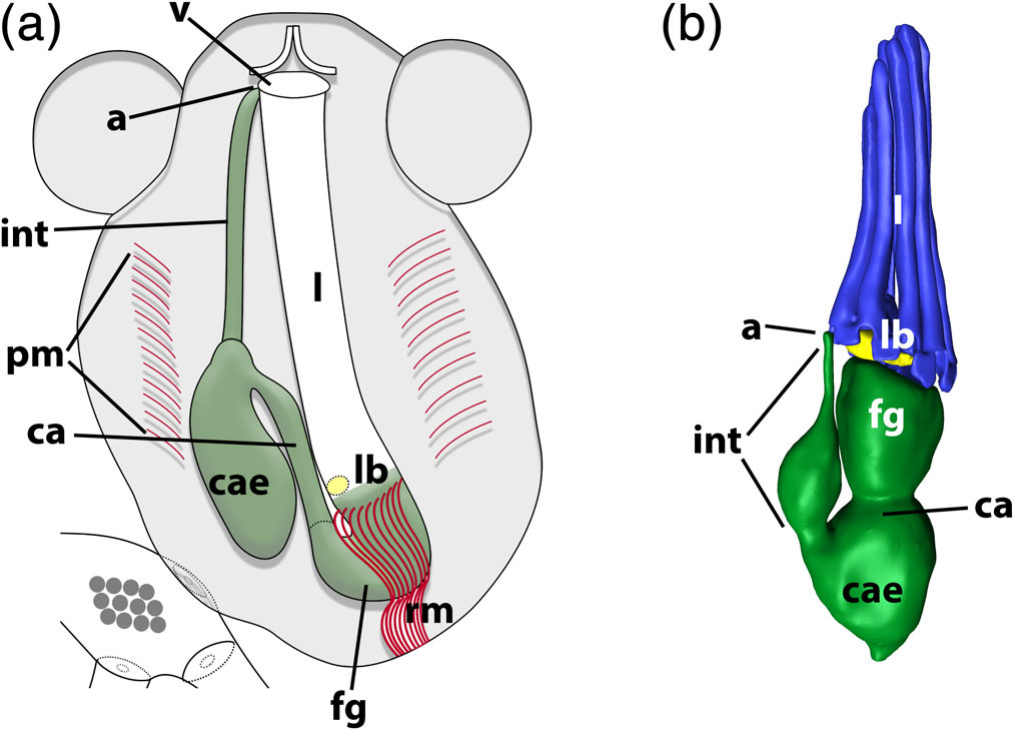
The position of the anus: (a) vestibular anus as exemplified by *H*. *expansa* (modified after Pröts, Wanninger, and Schwaha (2019)). (b) Lophophoral anus in *A*. *uraniae* (3D reconstruction, modified from Schwaha, Edgcomb, Bernhard, and Todaro (2019)). Abbreviations: a, anus; ca, cardia; cae, caecum; fg, foregut; int, intestine; l, lophophore; lb, lophophoral base; pm, parietal muscles; rm, retractor muscles; v, vestibulum

Ctenostome bryozoans show a high diversity of colonial forms that range from tightly encrusting, large erect, to boring, and include monomorphic to polymorphic taxa (Schwaha, 2019b). Recently, in an investigation on the polychaete‐tube inhabiting ctenostome *Hypophorella expansa*, it became evident that the location of the anus on the tentacle sheath was highly unusual and almost at the vestibular wall, close to the cystid wall (Figure 2a, Pröts et al., 2019), in contrast to other species that have their anus located at the lophophoral base (Figure 2b). Further preliminary analyses called for a much wider comparison of anal positions within bryozoans and especially ctenostomes. Hence, the main aim of this work is to analyse the position of the anus among ctenostome bryozoans and to assess whether positional variations might be functional adaptations, have occurred multiple times, and if the position of the anus is a systematically important character.

## 2 MATERIALS AND METHODS

Information on the zooidal position of the anus was taken from numerous ctenostome samples collected or received within the past decade and processed for histological analyses. In addition, data, especially from little‐known taxa, were taken from the literature (mainly drawings, illustrations).

For the creation of the comparative table, various zooidal characters were taken into account: anus position, tentacle number, peristome size, and zooidal density/colony form. Tentacle number is categorised as *low* with 8–10 tentacles, *medium* 10–20 tentacles, and *high* with more than 20 tentacles. Peristome size is more difficult to categorise as many zooids in several species essentially consist of a peristome (see Schwaha, 2019b). However, these were ranked from low when there is no or just slight peristomial elevation on the frontal zooidal side, to medium when this is approximately a third of estimated zooidal length, or high when exceeding that. Zooidal density was assigned as dense for regularly arranged colonies such those in colonies of *Alcyonidium* or *Flustrellidra*, to not dense when zooids are more spaced—the latter can be subject to change when the substrate becomes less available and growth can secondarily become very dense. A third category is dense tufts or rows, especially in polymorphic colonies that have stolons from which autozooids branch off.

## 3 RESULTS

**FIGURE 3:**
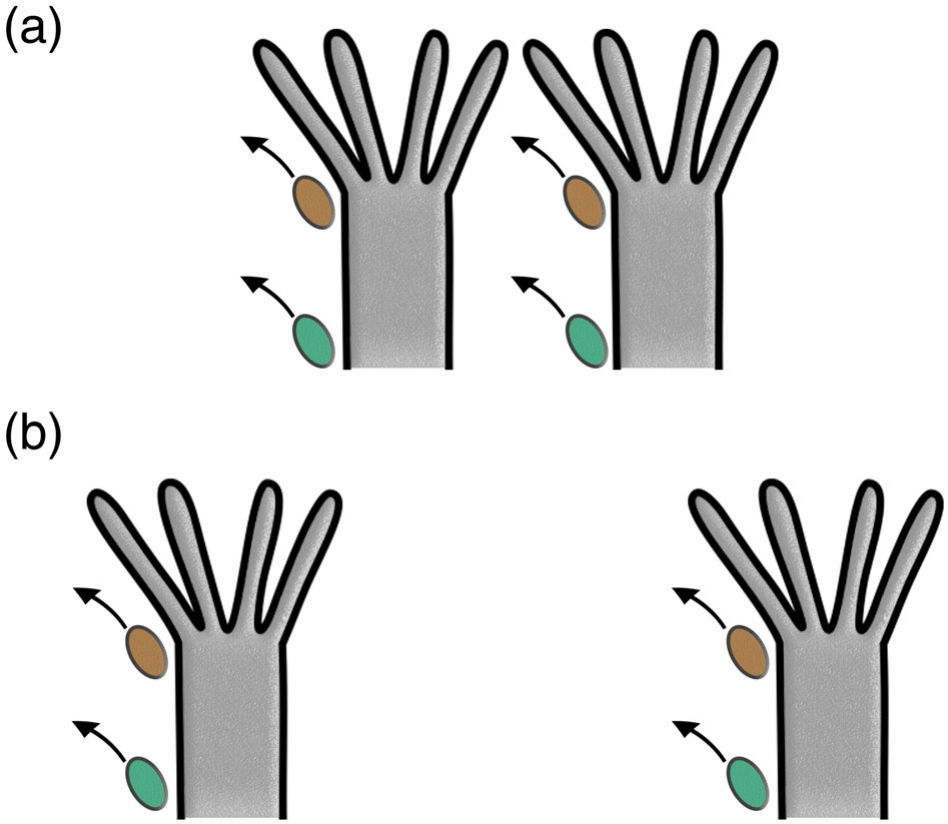
Schematic drawing of lophophore density and effects of a lophophoral and vestibular anus indicated by faeces in brown for the former and turquoise for the latter. (a) Dense zooidal arrangement. Defecation in the lophophoral anus will mostly likely interfere with feeding currents of neighbouring zooids, whereas the vestibular anus is less likely to do so. (b) “Colonial distancing” with zooids more widely spaced and less inter‐zooidal interactions

In general, two polarised, extreme positions of the anus can be distinguished among ctenostomes: the first is located close to the lophophoral base, closer to the ciliary feeding currents, the second is closer to the vestibular wall and thus further away from the lophophore (Figures 1 and 3). Accordingly, these are termed “lophophoral” and “vestibular” anus, respectively.

The distribution of anal positions is partially reflected in the traditional ctenostome superfamilies (Alcyonidioidea, Arachnidioidea, Hislopioidea, Paludicelloidea, Vesicularioidea, Victorelloidea, Walkerioidea, see Todd, 2000, Schwaha et al., 2019). Particularly striking is the ubiquitous presence of the vestibular anus among alcyonidioideans, whereas *Paludicella*, vesicularioideans, and victorelloideans show a lophophoral one. *Hislopia* and a few other species show an anus that terminates mid‐way on the tentacle sheath between the lophophoral base and vestibular wall. The latter condition might be more frequent among ctenostomes, but many illustrations and descriptions are not accurate enough for full evaluation. Arachnidioideans and walkerioideans show a mix of either lophophoral or vestibular positions. The distribution of other zooidal or colonial features such as tentacle number, peristome size, and colony arrangement does not reveal any specific pattern attributable to the location of the anus (Table 1). Especially, the presence of a distinct vestibular anus in alcyonidioidean and walkerioidean ctenostomes shows opposite zooidal and colonial features: dense versus rather non‐dense growth, high versus low tentacle numbers, often very high to low peristomial size.

**TABLE 1:** Anal position among ctenostome bryozoans with other zooidal and colonial characters. Based on literature or own unpublished observations

“Superfamily”	Family	Genus and Species	Anal position	Tentacle number	Peristomial size	Colony/density	Reference
Alcyonidioidea	Alcyonidiidae	*Alcyonidium* sp.	va	High	Mostly short to medium or even long in some species	Dense	d'Hondt (1983), Le Brozec (1955), and Schwaha (pers. Obs.)
	Flustrellidridae	*Flustrellidra hispida*	ma‐va	High	Short	Dense	Graupner (1930)
	Flustrellidridae	*Haywardozoon inarmatum*	va	Medium	Short	Dense	Hayward (1978) and d'Hondt (1983)
	Pherusellidae	*Pherusella* sp.	va	High	Short	Dense	Decker et al. (unpublished)
	Pherusellidae	*Pherusella tubulosa*	va	High	Long	Dense	Prouho (1892)
	Pachyzoidae	*Pachyzoon atlanticum*	va	High	Short	Dense	Schwaha (unpublished)
	Pachyzoidae	gen. and sp. nov.	va	High	Long	Dense	Schwaha (unpublished)
	Lobiancoporidae	*Lobiancopora hyalina*	va	High	Medium	Dense	Hayward (1985)
	Lobiancoporidae	*Bockiella angusta*	va	High	Medium	Dense	Silén (1942) and Hayward (1985)
	Sundanellidae	*Sundanella* sp.	va	High	High	Dense	Marcus (1941)
Arachnidioidea	Arachnidiidae	*Arachnidium fibrosum*	la	Medium	Short	Not dense	Schwaha (unpublished)
	Arachnidiidae	*Arachnidium hippothooides*	va	?	Short	Not dense	Hayward (1985)
	Arachnidiidae	*Arachnoidea raylankesteri*	va	Medium	Medium‐long	Not dense	Schwaha (pers. obs.)
	Nolellidae	*Nolella* sp.	la	Medium	Low/high^a^	Not dense	Calvet (1900)
	Nolellidae	*Nolella* cf. *papuensis*	la	Medium	Low/high^a^	Not dense	Harmer (1915)
	Nolellidae	*Nolella annectens*	la	Medium	Low/high^a^	Not dense	Gordon (1986)
	Nolellidae	*Nolella stipata*	la	Medium	Low/high^a^	Not dense	Osburn (1953)
	Immergentidae	*Immergentia* sp.	va	Low‐medium	Low	Not dense	Prenant and Bobin (1956)
	Immergentidae	*Immergentia suecica*	va	Low‐medium	Low	Not dense	Silén (1947)
	Immergentidae	*Immergentia californica*	ma	Low‐medium	Low	Not dense	Soule (1950)
	Immergentidae	*Immergentia philippinesis*	ma	Low‐medium	Low	Not dense	Soule (1950)
	Immergentidae	*Immergentia zelandica*	ma	Low‐medium	Low	Not dense	Soule (1950)
	Aethozoidae	*Aethozooides uraniae*	la	Medium	Low/high^a^	Solitary	Schwaha et al. (2019)
	Aethozoidae	*Franzenella limicola*	la	Medium	Low/high^a^	Solitary	Franzén (1960)
Hislopioidea	Hislopiidae	*Hislopia malayensis*	ma	Medium	Low	Dense	Schwaha and Wood (2011)
	Hislopiidae	*Hislopia corderoi*	ma	Medium	Low	Dense	Mane‐Garzon (1959)
	Hislopiidae	*Hislopia prolixa*	ma	Medium	Low	Dense	Hirose and Mawatari (2011)
	Hislopiidae	*Echinella placoides*	ma	Low	Low	Dense	Wiebach (1966)
Paludicelloidea	Paludicellidae	*Paludicella articulata*	la	Medium	Low	Not dense	For example, Allman (1856) and Prenant and Bobin (1956)
Vesciularioidea	Spathiporidae	*Spathipora comma*	la‐ma	Low	Low	Not dense	Soule (1950)
	Spathiporidae	*Spathipora mazatlantica*	la‐ma	Low	Low	Not dense	Soule and Soule (1976)
	Vesiculariidae	*Bathyalozoon foresti*	la	Low	Low	Not dense	d'Hondt (1976)
	Vesiculariidae	*Vesicularia fasciculata*	la	Low	Low	Dense tufts/rows	Osburn (1953)
	Vesiculariidae	*Amathia imbricata*	la	Low	Low	Dense tufts/rows	Reed (1988)
	Vesiculariidae	*Amathia caudata*	la	Low	Low	Dense tufts/rows	Annandale (1916)
	Vesiculariidae	*Amathia (Zoobotryon) verticillata*	la	Low	Low	Dense tufts/rows	Zirpolo (1933)
	Vesiculariidae	*Cryptopolyzoon* sp.	la	Low	Low	Dense tufts	Dendy (1888)
	Penetrantiidae	*Penetrantia brevis*	la	Low	Low	Not dense	Silén (1947)
	Penetrantiidae	*Penetrantia concharum*	la	Low	Low	Not dense	Silén (1947)
	Penetrantiidae	*Penetrantia irregularis*	la	Low	Low	Not dense	Gordon (1986)
	Penetrantiidae	*Penetrantia parva*	ma‐va	Low	Low	Not dense	Gordon (1986)
	Penetrantiidae	*Penetrantia densa*	ma	Low	Low	Not dense	Soule (1950)
	Penetrantiidae	*Penetrantia sileni*	ma	Low	Low	Not dense	Soule (1950)
Victorelloidea	Victorellidae	*Victorella pavida*	la	Low	Low/high^a^	Not dense	Braem (1951)
	Victorellidae	*Tanganella mülleri*	la	Low	Low/high^a^	Not dense	Braem (1951)
	Victorellidae	*Bulbella abscondita*	la	Low	Low/high^a^	Not dense	Braem (1951)
Walkerioidea	Hypophorellidae	*Hypophorella expansa*	va	Medium	Low	Not dense	Ehlers (1876) and Pröts et al. (2019)
	Aeverrillidae	*Aeverrillia setigera*	va	Low	Low	Dense tufts	Marcus (1937)
Walkerioidea	Walkeriidae	*Walkeria tuberosa*	la	Low	Low	Dense tufts	Harmer (1915)
Walkerioidea	Triticellidae	*Triticella minini*	va	Medium	Low	Dense tufts	Grischenko and Chernyshev (2015)
	Triticellidae	*Triticella* sp.	va	Medium	Low	Dense tufts	Hayward (1985)
Walkerioidea	Farrrelidae	*Farrella repens*	va	Medium	Low	Dense tufts	Marcus (1926)
Walkerioidea	Mimosellidae	*Bantariella tenuis*	la	Low	Low	Dense tufts	Harmer (1915)
	Mimosellidae	*Mimosella bigeminata*	la	Low	Low	Dense tufts	Harmer (1915)
	Mimosellidae	*Mimosella verticillata*	la	Low	Low	Dense tufts	Harmer (1915)
	Jebramellidae	*Jebramella angusta*	la	Low	Low	Dense tufts	Vieira, Migotto, and Winston (2014)
Incertae sedis	Pottsiellidae	*Pottsiella erecta*	la	Medium	High	Not dense	Braem (1940) and Smith, Werle, and Klekowski (2003)
Incertae sedis	Monobryozoidae	*Monobryozoon ambulans*	va	Medium	Low	Solitary	Remane (1938) and Gray (1971)
Incertae sedis	Panolicellidae	*Panolicella nutans*	la	Medium	High	Not dense	Jebram (1985)

Abbreviations: la, lophophoral anus; ma, mid‐positioned anus; va, vestibular anus.

a Strictly considered, the entire area containing the polypide is the peristomes, but on the comparison of vestibular wall size it remains low (see Schwaha (2019b)).

## 4 DISCUSSION

### 4.1 Location of the anus in bryozoans

This study shows that there is distinct variation in the position of the anus among ctenostome bryozoans. In comparison, the anus in non‐ctenostome bryozoans seems much more limited and restricted, showing little to no variation. As potential outgroups of gymnolaemaetes, phylactolaemate, and cyclostome bryozoans show that the anus is always lophophoral (e.g., Boardman, 1998; Mukai et al., 1997; Nielsen & Pedersen, 1979; Ryland, 1970), which indicates that this is the plesiomorphic, original condition. Hence, the vestibular anus among some of the ctenostome taxa is a derived condition, which potentially evolved several times. Little information is available for cheilostome bryozoans, and most descriptions/illustrations generally indicate the anus being located mid‐way between lophophoral base and vestibular wall (see, e.g., Calvet, 1900, Harmer, 1902, Marcus, 1937, 1938, 1939, Lutaud, 1977). However, it generally seems to be associated more with the lophophoral base in protruded zooids (McKinney, 1997). Cases with more a distally located anus have also been reported, however, among cheilostomes (see Lutaud, 1983; Nitsche, 1871).

### 4.2 A vestibular anus and the fixed anal position of phylactolaemates and cyclostomes

An important restriction in the position of the anus is constructional constraints in the organization of zooids in phylactolaemates and cyclostomes. In phylactolaemates, the position of the anus is fixed and has little possibility to be displaced. This is also connected to the fact that the epistomial coelom originates between the narrow space between the gut shanks and proceeds distally into the epistome above the mouth opening (Gruhl et al. 2009; Schwaha et al., 2019; Schwaha & Wood, 2011). Widening that space would probably affect the functionality and movement of the epistome as it would prevent fluid being easily channelled into the epistome.

Cyclostomes, in general, rarely protrude their lophophore much beyond the orifice or aperture, which has been considered a certain disadvantage concerning feeding competition when compared to the dominant cheilostomes (McKinney, 1988; McKinney & Boardman, 1985). This restricted range of protrusion is related to the morphological design of cyclostomes. They (and probably all stenolaemates) evolved a unique protrusion mechanism by detaching their peritoneal lining from the remaining body wall to form the so‐called membranous sac (Borg, 1926; Ernst, 2019; Schwaha et al., 2020). Proximally, the membranous sac is connected to the cystid wall where the retractor muscles attach to the skeleton, whereas distally so‐called attachment organs and ligaments are frequently found in the apertural area (Boardman, 1998; Ernst, 2019). These restrict movement of the polypide in respect to the cystid.

Gymnolaemates, on the other hand, have the possibility to shift their anus in their more flexible and protrusible poylpipes (McKinney 1988; Winston, 1978). However, the mechanism causing the shift in the anus of ctenostomes remains unclear as the current study indicates that there do not seem to be any distinct zooidal or colonial traits correlated with the position of the anus. The general tendency to a higher polypide protrusion capability is linked to a more efficient feeding mechanisms and flexibility, allowing coordinated colonial integration (e.g., Shunatova & Ostrovsky, 2001, 2002; Winston, 2019).

### 4.3 What we can learn from ctenostome anuses?

Most ctenostomes are only studied as preserved and generally always retracted forms. As previously stated, the introvertable area of individual zooids has a high range with respect to the vestibular wall (Schwaha, 2019a, 2019b). The vestibular wall can be quite extensive in several species and might even exceed the length of the tentacle sheath if not the polypide itself. This is particularly evident in many alcyonidioidean species that always have a vestibular anus. Consequently, mere introversion of the tentacle sheath has little effect in protruding the tentacle crown. As a consequence, the vestibular wall requires extensive inversion (see also Schwaha, 2019a, 2019b), especially among species with a vestibular anus in order to defecate into the open water column and not into the vestibulum. Given the long length of certain vestibular walls, this implies that lophophores must extend quite far from the zooidal orifice into the water column in live, protruded zooids. This is, unfortunately, little studied so far and would require live observations. However, it shows that the position of the anus has some implications of how live colonies might function when we only have preserved material at hand (especially of deep‐sea ctenostomes).

In general, little is known on the effects, consequences and differences of vestibular wall size and inversion in protruded versus retracted zooids. This is an important issue to address in several ctenostome genera in the future, in particular because the distance of the vestibular anus of a retracted polypide does not necessarily correspond to the situation in protruded ones. While the vestibular wall is usually lacking musculature and is lined by the same cuticle as the remaining cystid wall, the tentacle sheath always carries longitudinal muscle fibres and thus can shorten (Schwaha & Wanninger 2018, Schwaha, 2019a).

As already mentioned, the position of the anus does not seem to correlate with colony morphology or zooidal arrangement and hence does not indicate any functional advantages in the feeding process. Instead, the occurrence, in particular, of a vestibular anus appears in closely related taxa, that is, all alcyonidioideans, along with other soft‐tissue characters, aids in characterizing this clade (see Schwaha, 2019a, 2019b; Schwaha & Wanninger 2018). Likewise, the walkerioidean genera *Triticella*, *Farrella*, and *Aeverrillia* are often stalked, stolonate forms that also share a vestibular anus, whereas other walkerioideans, such as *Mimosella* and *Walkeria*, have a lophophoral anus.

Possibly striking as a clear aid in further addressing its phylogenetic position is the vestibular anus found in *Monobryozoon* (Table 1, Remane, 1936, 1938), which supports a closer relationship to the Alcyonidioidea. Likewise, the vestibular anus is another confirmation that *Sundanella* also belongs to this taxon and is not associated with victorellid ctenostomes (see also Braem, 1939; Schwaha, 2019b).

Other ctenostome taxa show a variety or mosaic concerning their anal position. Boring bryozoans are distributed in four different families which according to their colony morphology and zooidal details probably evolved at least twice independently (Jebram, 1973, 1986; Schwaha, 2019b). Their anal distribution currently represents quite a mosaic of lophophoral to vestibular anuses (Table 1).

The small taxon Hislopioidea with fewer than 10 species shows a mid‐positioned anus, but similar to the vestibular anus, currently has little functional or evolutionary explanation. Particular lack of data still remains for the “Arachnidioidea,” a heterogeneous clade, which almost completely lacks any detailed soft morphological studies (Schwaha, 2019b), although first studies will start to emerge in the near future (Table 1).

### 4.4 Consequences of the position of the anus: Feeding and defecation

Keeping zooids in a colony in close proximity enhances the capacity of suspension feeding and, with respect to the high competition faced by other benthic suspension feeders, is a vital character for numerous bryozoans. Colonial density also has its drawbacks: feeding currents of neighbouring zooids interact and certain adaptations are necessary for creating exhalant currents for nutrient‐depleted water (Shunatova & Ostrovsky, 2001, 2002, Winston, 2019, see also Figure3). In a similar manner, defecation interacts between closely spaced zooids (McKinney, 1997, Figure 3A). Colonial distancing has advantages by minimising interactions of feeding or defecation events (Figure3). However, as previously mentioned, distancing lowers feeding currents with isolated zooids (Winston, 1979) and seems to be a clear selective disadvantage.

Particularly among densely aggregated zooids, faecal pellet disposal is an important aspect of bryozoan coloniality (McKinney, 1997). This is particularly evident in most cheilostomes, where also most observational data are present (McKinney, 1997; Shunatova & Ostrovsky, 2001, 2002; Winston, 2019). Numerous colonies create chimneys for excurrent, nutrient depleted water currents that also serve for faecal pellet disposal (see references above). This is present among phylactolaemates (Mukai 1999), recent cyclostomes and cheilostomes (Shunatova & Ostrovsky, 2002) and also is evident among fossil stenolaemates (often by the presence of so‐called monticules; Ernst, 2019). Such chimneys usually cover areas devoid of autozooids. In cheilostomes lacking such specific areas, different strategies commonly apply for waste removal such as directional colonial movements effectuated by enlarging, often asymmetrically arranged, lophophores towards the colony margin, or concerted particle removal by “catch and play” behaviour. In the latter, undesirable or unpalatable particles are removed from individual zooids by ciliary reversal of the lophophore. Such particles are thus continuously transported from one zooid to its neighbour until to the colony margin (Shunatova & Ostrovsky, 2001; Winston, 2019).

Few observations have been historically conducted on defecation in bryozoans (see Best & Thorpe, 1987; Silén, 1944; Winston 1977). Most of these are on calcified taxa, whereas the few ctenostome observations were conducted on vesicularioideans. Four different pathways for faecal pellet removal have been recognized among cheilostomes (and a few ctenostomes with a lophophoral anus; McKinney, 1997). Two of these (Pathways 1 and 2) involve faecal pellets entering the lophophore, whereas the other two (Pathways 3 and 4) redirect faecal pellets without entering the circular lophophore. In theory, the displacement of the vestibular anus from the lophophore prevents faecal pellets from entering it (see Figure 3). Also, it would appear improbable for the faecal pellet to be transported on the outer margin of the lophophore (as in Pathway 4, McKinney, 1997). Hence, other pathways probably occur among ctenostomes with vestibular anuses.

In contrast to colonial distancing, a vestibular anus would prevent faecal interference with feeding currents in tight zooidal arrangements (Figure 3), but implies that faeces would aggregate on the colony surface. However, faecal accumulations on the colony surface do not seem to be a regular condition among such colonies. Zooids of erect colonies or those that grow on the underside of substrates naturally do not face such a problem. Likewise, frequent water movements (e.g., in epiphytic colonies) also aid in pellet removal, and zooidal intra‐colonial interaction also has cleaning purposes of the colony (Shunatova & Ostrovsky, 2001, 2002). In sum, there remain numerous open questions concerning defecation and colonial integration. Future observations of live animals are required and should clarify which pathways might be at work in various ctenostomes and whether details in polypide anatomy show distinct differences.

The studied ctenostomes such as *Bowerbankia*/*Amathia*(e.g., Winston 1977) that have a typical lophophoral anus were considered to follow defecation Pathway 1 as described for several cheilostomes (McKinney, 1997). Although direct evidence has not been reported for a vestibular anus in the alcyonidioidean *Flustrellidra hispida* (in contrast to all others of the clade), defecation of individuals occurs in 65% of all cases when polypides protrude or retract (Best & Thorpe, 1987), which indicates that polypide movements might be important if not necessary for such taxa.

## 5 CONCLUSION

This study shows that there is a general variability in the location of the anus among ctenostomes and also underlines how little we still know about many basic features of bryozoans in general. Numerous issues remain open for future studies, including: morphology of the digestive tract and correlation with the position of the anus, general diversity of cheilostome guts, and study of live animals, especially ctenostomes with vestibular anuses. Along with other increasing data on soft tissue morphology (Schwaha, 2019a, 2019b; Schwaha et al., 2020), the position of the anus is an important character for phylogenetic inferences as it does not seem to correlate with zooidal or colonial characters. Molecular trees of ctenostomes just start to appear (e.g., Waeschenbach, Vieira, Reverter‐Gil, Souto‐Derungs, Nascimento, & Fehlauer‐Ale, 2015) and once a new and more complete phylogenetic tree of ctenostome bryozoans is available, it should become clearer how often a vestibular anus has evolved.

## AUTHOR CONTRIBUTION

Thomas Schwaha: Conceptualization; data curation; formal analysis; investigation; writing‐original draft; writing‐review; and editing.

## Data Availability

The data that support the findings of this study are available from the corresponding author upon reasonable request.
